# Tissue‐Resident Macrophages in Cancer: Friend or Foe?

**DOI:** 10.1002/cam4.70387

**Published:** 2024-11-04

**Authors:** Jianhua Chi, Qinglei Gao, Dan Liu

**Affiliations:** ^1^ Department of Obstetrics and Gynecology National Clinical Research Center for Obstetrics and Gynecology, Tongji Hospital, Tongji Medical College, Huazhong University of Science and Technology Wuhan China; ^2^ Key Laboratory of Cancer Invasion and Metastasis (Ministry of Education), Hubei Key Laboratory of Tumor Invasion and Metastasis Tongji Hospital, Tongji Medical College, Huazhong University of Science and Technology Wuhan China

**Keywords:** cancer immunology, tissue‐resident macrophages, tumor microenvironment, tumor‐associated macrophages

## Abstract

**Introduction:**

Macrophages are essential in maintaining homeostasis, combating infections, and influencing the process of various diseases, including cancer. Macrophages originate from diverse lineages: Notably, tissue‐resident macrophages (TRMs) differ from hematopoietic stem cells and circulating monocyte‐derived macrophages based on genetics, development, and function. Therefore, understanding the recruited and TRM populations is crucial for investigating disease processes.

**Methods:**

By searching literature databses, we summarized recent relevant studies. Research has shown that tumor‐associated macrophages (TAMs) of distinct origins accumulate in tumor microenvironment (TME), with TRM‐derived TAMs closely resembling gene signatures of normal TRMs.

**Results:**

Recent studies have revealed that TRMs play a crucial role in cancer progression. However, organ‐specific effects complicate TRM investigations. Nonetheless, the precise involvement of TRMs in tumors is unclear. This review explores the multifaceted roles of TRMs in cancer, presenting insights into their origins, proliferation, the latest research methodologies, their impact across various tumor sites, their potential and strategies as therapeutic targets, interactions with other cells within the TME, and the internal heterogeneity of TRMs.

**Conclusions:**

We believe that a comprehensive understanding of the multifaceted roles of TRMs will pave the way for targeted TRM therapies in the treatment of cancer.

## 1 Introduction

Macrophages are innate immune cells found in non‐immune organs [1]. Macrophages can polarize and induce diverse functional phenotypes in reaction to external stimuli. Two decades ago, Mills et al. proposed a binary classification of macrophage polarization into M1 and M2 phenotypes [2]. “M1‐like” phenotype is typically pro‐inflammatory, recognized as associated with anti‐tumor macrophages. Conversely, “M2‐like” macrophages are characterized as anti‐inflammatory and pro‐cancer. However, this paradigm has been further refined. Studies have shown that macrophages support tumor growth, angiogenesis, immunosuppression, and metastasis in tumors [3]. Co‐expression of both M1 and M2 gene signatures has been identified in macrophage subsets across nearly all cancer types [4, 5, 6]. Macrophages can be grouped into subpopulations using new technologies [7]. Tissue‐resident macrophages (TRMs) and circulating monocytes provide the pool of tumor‐associated macrophages (TAMs) [8]. TRM‐derived TAMs closely resemble gene signatures of normal TRMs [9, 10]. Besides, TRM‐derived TAMs are distinct from macrophages derived from hematopoietic stem cells (HSCs) or monocytes in terms of genetics, development, and function [11, 12]. Although monocytes can replace TRMs when the tissue niche is unoccupied [7, 13], TRMs cannot fully be substituted by the recruitment of bone marrow‐derived macrophages (BMDMs) [12]. TRMs can induce pro‐tumorigenic or anti‐tumorigenic effects through phagocytosis and the release of diverse factors to regulate the immune response [8, 9, 14, 15, 16, 17, 18]. The functions of TRMs in cancer are organ‐specific [19]. TRMs are involved in the initial stages of cancer development and the formation of micro‐metastases, whereas BMDMs are recruited during later phases [19]. The proportion of recruited macrophages and TRMs is essential for investigating various disease conditions [20]. Nonetheless, discrepancies in repopulation patterns have been observed between recruited macrophages and TRMs after treatment or clearance strategies in various cancer models [9]. The life span, rate of replacement, and replenishment of monocytes for TRMs differ across various organs, thereby limiting TRM studies [8]. Besides, the precise functions and roles of TRMs in cancer are unclear.

This review explores the multifaceted roles of TRMs in cancer, presenting insights into their origins, proliferation, the latest research methodologies, their impact across various tumor sites, their potential and strategies as therapeutic targets, interactions with other cells within the tumor microenvironment (TME), and the internal heterogeneity of TRMs.

## 2 Necessity of Distinguishing TRMs From Monocyte‐Derived TAMs and the Latest Research Methods

Distinguishing TRMs from monocyte‐derived macrophages within TAM populations is fundamental to TRM research. Recent technological advancements have made this distinction achievable. TRMs and monocytes‐derived TAMs exhibit different transcriptional profiles and functions [21]. Gomez Perdiguero et al. found that monocyte‐derived macrophages exhibit profibrotic transcriptional profiles, while resident macrophages with specific molecular characteristics support tumor progression [22]. Also, resident peritoneal macrophages with reduced mTORC1 activity, elevated levels of arginase 1 (Arg1) expression, and mitophagy activity support tumor advancement in murine models lacking chemokine receptor C‐C motif chemokine receptor (CCR) 2. In contrast, macrophages derived from monocytes do not demonstrate this tumor‐promoting effect [23]. Qie et al. [24] evaluated 10 primary macrophage populations from seven mouse tissues and confirmed that IL‐18 can be used to distinguish the molecular markers and cell function characteristics between macrophages residing in tissues and macrophages recruited in lungs and livers. Lavin et al. [25] revealed that lineage‐ and tissue‐specific transcription factors regulate chromatin specification in TRMs. Macrophages cannot be solely identified based on their origin alone as both intrinsic and extrinsic factors interact to shape their characteristics, influenced by the timing of recruitment and environmental cues within their tissue microenvironment [26].

TRMs can be distinguished and monitored using various techniques. Besides, the advancements in technology have improved the research on macrophages, especially on developmental origin, niche, functions, and diversity [27]. Some cutting‐edge tools, including single‐cell RNA sequencing (scRNA‐seq), mass cytometry, imaging mass cytometry, and multiplexed high‐resolution imaging techniques, play a crucial role in addressing unresolved queries related to distinct functions, tumor regions, and the roles of different developmental origins of TAMs [9]. Saylor et al. reported a method, PLEXODY, which consists of five myeloid cells and macrophages related fluorescently tagged antibodies [28]. Guillot et al. also developed an immunohistochemistry technique for the rapid differentiation between monocyte‐derived macrophages and Kupffer cells in mouse samples [29]. Furthermore, CO‐Detection by indEXing (CODEX) multiplexed imaging platform can achieve spatial location analysis of immune cells, including TRMs within tumors [30, 31]. Notably, microglia can be distinguished from recruited macrophages through various methods [32]. Schulz and Sevenich [33] summarized recent findings on the molecular level similarities and differences of central nervous system (CNS)‐resident macrophages with diverse sources. Spectral flow cytometry allows more fluorochromes and captures the autofluorescence of samples or cell populations, such as macrophages [34]. Bourdely et al. evaluated macrophages in skin and cutaneous squamous cell carcinoma using spectral flow cytometry [35]. Differences among species impact the extent of cell infiltration from peripheral sources. Recent methodologies, such as lineage tracing models and scRNA‐Seq, can provide unbiased assessments of peripheral infiltration and disease‐specific effects on myeloid subpopulations [36]. Furthermore, humanized mice and in vitro culture systems can be used to study human macrophages in native tissue microenvironments [37, 38]. Aktories et al. developed an organotypic primary cell culture method to sustain monocultures of macrophages without prior purification, providing a screening platform for diverse organ‐specific macrophages [39]. The application of 3D macrophage models can enhance disease treatments [40]. Some of variability identified through scRNA‐Seq or other cell isolation techniques may not accurately represent biological processes in vivo. Authentic variations in macrophage phenotypes can be linked to their specific locations within tissue subdomains or life histories. However, single‐cell data demonstrating the functional significance of markers that define cellular heterogeneity are lacking. Although the functional significance of markers that define cellular heterogeneity can be determined through the deletion of targeted genes using Cre recombinase, this method may introduce potential artifacts [7]. In addition, TAM subsets cannot be easily identified as monocyte‐derived macrophages can adopt certain TRM markers and functions during tissue damage and tumor development.

## 3 The Origin and Proliferation of TRMs

**FIGURE 1:**
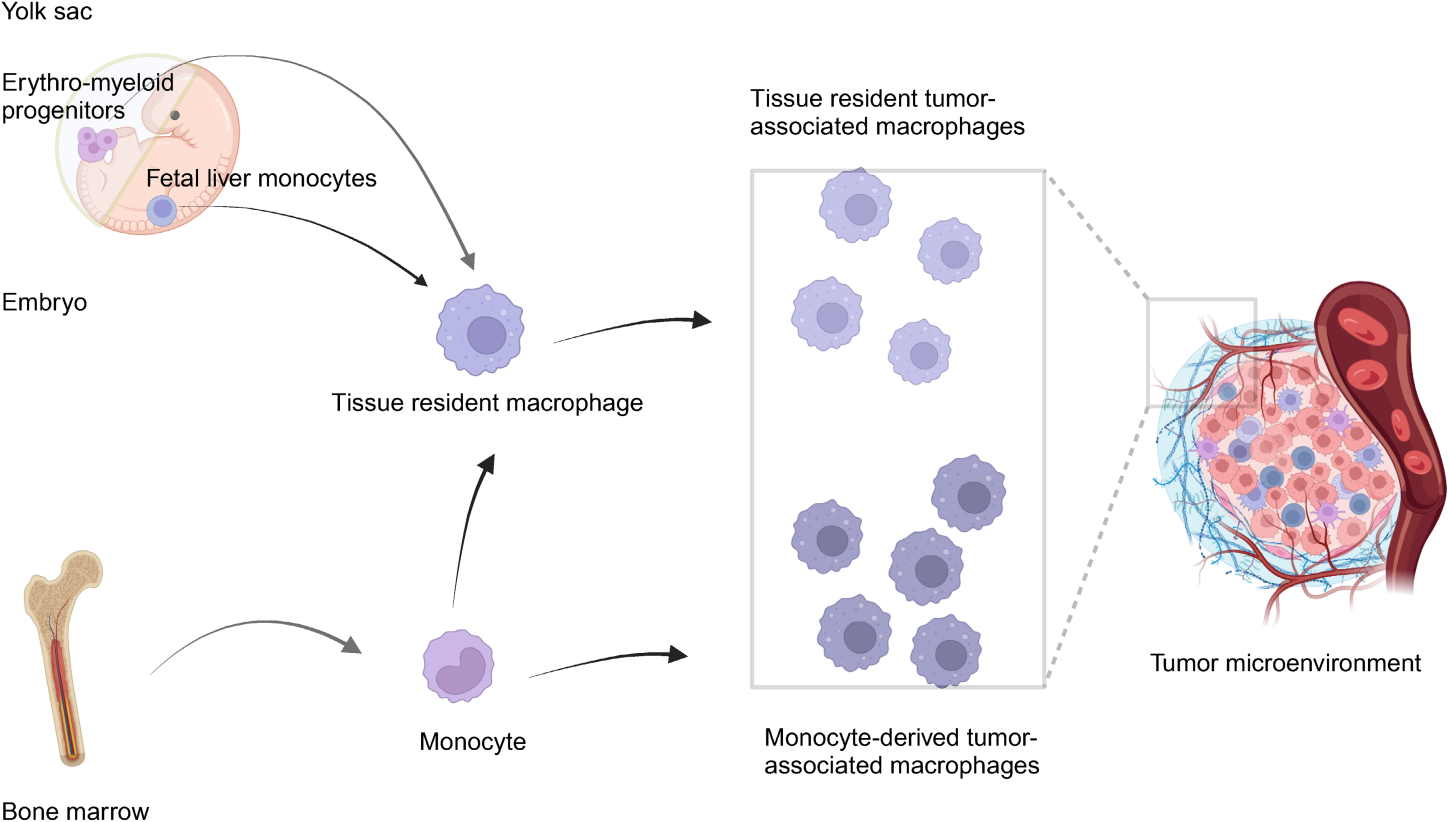
Source of tumor‐associated macrophages.

Recognizing the features of origin and proliferation is the cornerstone of comprehending the functions of TRMs. Macrophages originate from various lineages. TRMs are thought to originate directly from yolk sac progenitors without the involvement of bone marrow intermediates [41], self‐renewing locally and independently of adult hematopoiesis [42, 43, 44, 45, 46]. Sheng et al. also showed that TRMs are derived from HSCs undergoing distinct development pathways separate from yolk sac or fetal liver in embryo [47, 48]. Hoeffel et al. demonstrated that EMPs produced in yolk sac give rise to major precursors of microglia. The fetal monocytes inoculating into embryonic tissues then differentiate into other adult TRMS [49]. Zhao et al. identified two primitive and one definitive EMPs subpopulations originating from the yolk sac, which are responsible for the generation of microglia and other TRMs, respectively [50]. In conclusion, TRMs originate from three precursors at various locations and time points (Figure 1): early yolk sac macrophages, fetal liver monocytes, and bone marrow‐derived monocytes [51]. The specific microenvironment of each location, known as the “niche of residence,” provides necessary signals for the development of functional resident macrophages from any precursor in a time‐dependent manner [52]. Bain et al. [53] showed that resident F4/80^(hi)^GATA6 (+) macrophages exhibit longevity, non‐stochastic self‐renewal ability, and retain of embryonic origin cells in mice for at least 4 months. TLF macrophages, identified by the presence of T‐cell immunoglobulin mucin‐4 (Tim4 or TIMD4), lymphatic vessel endothelial hyaluronan receptor 1 (LYVE1), and folate receptor beta (FOLR2), are derived from both yolk sac and fetal monocyte precursors and maintained through self‐renewal with minimal monocyte input. In contrast, CCR2 (TIMD4^++−^LYVE1^−^FOLR2^−^) macrophages are predominantly replenished by monocytes. Although MHC‐II^hi^ macrophages (TIMD4^−^LYVE1^−^FOLR2^−^CCR2^−^) are replenished by monocytes, they are not continuously replaced. Monocyte‐derived macrophages maintain the resident macrophage population until a specific upper limit is reached [54].

TRMs produce TAMs. Ma et al. identified seven TAM subsets which are preserved in almost all cancer types. Notably, RTM‐TAMs are enriched both in adjacent tissues and within tumors [4]. The regulation of TRMs proliferation is complex. Over 100 transcription factors and cis‐regulatory regions are related to the identity of TRMs [55]. F4/80^hi^MHCII^low^ macrophages are the predominant subset of tumor‐resident macrophages in colon adenomas of Apc^Min/+^ mice as the tumor advances. These sustain their population primarily through mechanisms not reliant on bone marrow input [56]. Soluble factors may induce macrophage self‐renewal [57]. Purine nucleoside adenosine, originating from tumors, controls macrophages self‐renewal through the PI3K/Akt and MEK/ERK signaling pathways [58]. Soluble form of triggering receptor expressed on myeloid cells 2 (TREM2) promotes microglial survival by activating PI3K/Akt, ERK1/ERK2, and p38 signaling pathways [59, 60]. Oh et al. [61] showed that mTORC2 inhibition enhances GATA6 expression through FOXO1, thus influencing the differentiation programming of peritoneal macrophages. Therefore, mTORC2 activation can be used to distinguish TRM proliferation from M2 macrophage differentiation. Sakai et al. [62] demonstrated that the induction and maintenance of Kupffer cell characteristics occur due to the combined interaction of the Notch ligand DLL4 with transforming growth factor‐b (TGF‐β) family ligands and endogenous LXR ligands.

## 4 TRMs Biomarkers

Macrophages exhibit significant heterogeneity, indicating that understanding the biomarkers associated with TRMs is crucial for distinguishing these cells with other macrophages and investigating their functions. Macrophages in mice and humans are labeled as CD11b^+^F4/80^+^CSF‐1R (CD115)^+^Gr1^−^ and CD68^+^CD163^+^CD16^+^CD132^+^ CD115^+^, respectively [63]. Notably, resident microglia and monocyte‐derived infiltrating BMDMs have different CD45 expression levels [64]. Resident microglia are the major source of TAMs in primary brain tumors [65, 66]. Infiltrating BMDMs account for 85% of the total TAM in glioblastomas [67]. However, microglia and BMDM‐derived macrophages account for 60% and 40% in glioma mouse model, respectively [68]. *Itga4* (Cd49d) is a specific marker of monocyte‐derived macrophages in many brain malignancies [69]. Most colonic stromal immune cells are resident macrophages. These cells express sialic acid‐binding immunoglobulin‐like lectin‐7/−9, with high level of CD68/CD163 and low level of CD14/CD89 [70]. Tim‐4, CD16, CD206, CD163, and CRIg are some possible markers of resident macrophages in ascites of ovarian cancer patients [71, 72]. Ms4a3 is highly expressed in granulocyte–monocyte progenitors (GMPs) but not in Ly6C^high^ (or Ly6C^low^) monocytes, or any mature TRM populations at homeostasis [73]. Larionova et al. [74] identified some biomarkers (stabilin‐1, CD163, CD206, CD204, and MARCO [macrophage receptor with collagenous structure]) crucial for functional TAM polarization. Recent studies have shown that TAM populations with highly expressed HES1, FOLR2, CD163, and CD206, are partially derived from TRMs, while other TAM subsets are derived from monocytes [4, 10, 75]. VSIG4, V‐set, and immunoglobulin domain containing 4, also known as CRIg, is a complement receptor. hVSIG4 protein is selectively expressed in a subpopulation of TRMs, such as the peritoneum and Kupffer cells [76, 77, 78, 79, 80, 81]. Liao et al. demonstrated that the growth of murine lung cancer cells is significantly inhibited in VSIG4‐deficient mice [82].

## 5 Role of TRMs in Cancer: Friend or Foe?

TRMs are an essential component of TAMs. Certain subsets of TRMs exhibit a high expression of M2 markers, including CD206 and CD163 [4, 10, 71, 72, 75]. M2 polarization seems to be the predominant state for tissue‐resident macrophages, yet M2 and M1 macrophages are capable of interconversion in response to specific environmental cues [83]. Nevertheless, the significant heterogeneity and diverse functions observed among tissue‐resident macrophages indicate that these cells cannot be simplistically classified as merely “M1” or “M2” when present within tissues [84]. The multifaceted roles of TRMs in various cancers are highlighted below (Table 1).

**TABLE 1:** Role of TRMs in cancers.

Author	Cancer type	Sample resource	TRM‐TAMs function	MoD‐TAMs function	Depletion TRMs	Refs.
Loyher	Lung cancer	Mice	Tumor cell growth	Tumor spreading	Anti‐VEGF treatment and chemotherapy combination hindered both Res and Mod‐TAMs reconstitution without affecting monocyte infiltration, treatment efficacy improved	[85]
Casanova‐Acebes	Early NSCLC	Human, mice	Promotes tumor invasiveness and induce Treg cell response		Depletion of TRMs reduce invasiveness and growth	[86]
Prieto	Lung cancer	Kras‐driven lung cancer mice model.	Cytotoxic T‐cell responses were suppressed by alveolar macrophages		Attenuates adenoma development and progression	[87]
Sharma	Metastasis to the lungs	Mice	Immunosuppression		Reduced lung metastatic burden	[88]
Tapmeier	Metastasis to the lungs	Mice melanoma B16F10 model	Pro‐inflammatory and anti‐tumor	From pro‐inflammatory to later tumor‐promoting profile		[89]
Shang	Metastasis to the lungs			Suppress lung metastasis		[90]
Kramer	Metastasis to the lungs	Solid tumor models	Heightened lung metastasis			[91]
Vanderborght	Liver cancer	Mice model			No impact on HCC pathogenesis	[13]
Wen	CRC liver metastases	Orthotopic murine model	Bimodal role in tumor growth		Increase tumor burden at early, decrease liver tumor load at late stage	[92]
Hossain	Liver metastases	Mice	Metastatic growth and tumor recurrence			[93]
Dumas	Microglia	Human, mice	Immune evasion and tumor growth			[94]
Wang	Microglia	Rat			Glioma progression	[95]
Zhu	Pancreatic carcinoma		Pro‐fibrotic transcriptional profile			[14]
Dong	PDAC	Human	GLUL‐SQSTM1‐RTM, a positive regulator of immunity			[96]
Liao	PDAC	Postoperative pancreatic patients	High infiltration of CXCR4 macrophages linking with poor overall and disease‐free survival			[97]
Zhang	PDAC	Both humans and mice chemotherapy‐treated samples	Promote fibrosis and immunosuppression, poor clinical outcomes			[98]
Hirano	Breast tumor	Mice			Decreases tumor growth and TAMs infiltration. Reduce recurrence and distant metastases. Enhance chemotherapy efficacy	[18]
Nalio Ramos	Breast cancer	Human	FOLR2 macrophages, improves patient survival			[99]
Gunnarsdottir	Breast cancer	Human, mice	CD169 TRMs in lymph nodes, beneficial prognosis	CD169 Mo‐M in primary tumor, immunosuppression		[100]
Krishnan	Ovarian cancer	Mice	Enhances cancer cell migration and colonization in omentum		Reduces ovarian cancer colonization	[101]
Etzerodt	Ovarian cancer	Mice	CD163+ Tim4+ TRMs, promote metastasis		Prevent tumor progression and metastasis	[102]
Zhang	Ovarian cancer	Mice	LYVE1^hi^ mesothelial macrophages driving tumor growth		Ablation of LYVE1^hi^ macrophages significantly reduced syngeneic epithelial ovarian tumor growth	[103]
Joshi	Ovarian cancer	Mice	TIM4+ LPMs contributes to immune surveillance of the peritoneum		TIM4 deletion blunts induction of early anti‐tumoral effector CD8 T cells and accelerates tumor progression	[104]
Katholnig	CRC	Human, mice	High SPP1 macrophages, worsens prognosis			[105]
Liu	CRC	Human	FOLR2 TRMs, unfavorable prognosis			[106]
Ohnishi	CRC	Human	CD169 (+) TRMs in RLNs, longer overall survival			[107]

Abbreviations: AM, alveolar macrophages; CRC, colorectal cancer; FOLR2, folate receptor beta; GLPMs, GATA6 large peritoneal macrophages; HCC, hepatocellular carcinoma; KC, Kupffer cells; MDSC, myeloid‐derived suppressor cells; MGTRMs, mammary gland tissue‐resident macrophages; NSCLC, non‐small cell lung cancer; PDAC, pancreatic ductal adenocarcinoma; RLNs, regional lymph nodes; rMφs, resident macrophages; TAM, tumor‐associated macrophages; TNBC, triple‐negative breast cancer; TRM, tissue‐resident macrophages.

### 5.1 TRMs Contribute to Tumor Progression, Metastasis, and Recurrence, Resulting in a Poor Prognosis

TRMs are essential in the early‐stage lung tumors [108]. Loyher et al. [85] found that tissue‐resident interstitial macrophages (Res‐TAMs) are associated with tumor cell growth. Alveolar macrophages accumulate early in neoplasia and promote tumorigenesis by suppressing cytotoxic T‐cell responses in mouse model of Kras‐driven lung cancer [87]. Alveolar macrophages are also involved in pre‐metastatic niche (PMN) formation. Sharma et al. [88] demonstrated that alveolar macrophages accumulate in lung before breast cancer metastasis via complement C5a receptor‐mediated proliferation. Moreover, alveolar macrophages promote cancer metastasis in mouse lungs by suppressing anti‐tumor T cells. Shang et al. [90] discovered that deficiency of CXCR3/TLR4 inhibits the expression of CCL12 in alveolar macrophages, thus hindering monocytic myeloid‐derived suppressor cells (mo‐MDSCs) recruitment to PMN, thereby mitigating lung metastasis. Kramer et al. [91] indicated that *β*‐catenin expression in alveolar macrophages enhances metastasis in solid tumor models.

Liver macrophages include both self‐renewing resident Kupffer cells and monocyte‐derived macrophages [109]. In a study using scRNA‐Seq of murine non‐alcoholic steatohepatitis (NASH) livers, proliferating macrophages expressed Spp1, which encodes the protein osteopontin (OPN), indicating poor outcome in HCC [110]. A high density of total CD68 macrophages in the tissue samples is related to poor prognosis in HCC patients [111]. Hossain et al. discovered that GATA6‐related large peritoneal macrophages (GLPMs) promote the growth of liver metastasis by breaching the visceral mesothelium of liver [93].

Microglia and perivascular macrophages are resident myeloid cells in CNS [112]. Microglia can activate the mTOR pathway in murine glioma models, leading to poor survival outcomes [94, 113, 114]. Wang et al. discovered that reducing CD200 levels in CD200‐abundant glioma cells can enhance the formation of a TME characterized by activated microglia, thereby facilitating glioma development [95]. VSIG4 expression is associated with poor prognosis in patients with high‐grade glioma [115], advanced gastric cancer [116], and ovarian cancer patients [23].

TRM‐induced fibrosis promotes the development and progression of pancreatic carcinoma [117]. Depletion of TRMs can reduce fibroblast accumulation and decrease tumor burden induced by pancreatitis [118]. Liao et al. investigated the link between high infiltration of CXCR4 macrophages with poor overall and disease‐free survival in pancreatic cancer patients [97]. Zhang et al. revealed that proliferating resident macrophages are associated with unfavorable clinical outcomes by avoiding chemotherapy through specific metabolic mechanisms [98].

Macrophages play a crucial role in normal mammary gland development and breast cancer progression [119, 120]. Although TAM has potential tumoricidal activity, they can be re‐polarized toward a pro‐tumor phenotype by the tumor [121, 122]. TAM exhibit similarities to ductal macrophages in terms of gene expression profiles and functions related to revascularization and tissue remodeling [123, 124, 125]. Mammary gland TRMs (MGTRMs) are the predominant stromal cells in early triple‐negative breast cancer (TNBC) before angiogenesis. MGTRMs in the TNBC promote recurrence and distant metastases [18]. Gunnarsdottir et al. [100] also showed that CD169 resident macrophages are associated with TLSs and Tregs, which are correlated with worse prognosis in primary breast cancer.

Macrophages are highly prevalent within the ovary and facilitate tumor cell colonization in the omentum [126]. Chemokine ligands secreted from macrophages increase migration and colonization of ovarian cancer cells in the omentum. Furthermore, these macrophages can interact with CCR1 [101]. Embryonically derived resident macrophages (Tim‐4, CD163, and LYVE‐1 in peritoneal fluid, omentum, and peritoneum, respectively) can intraperitoneally promote ovarian cancer progression in mice. Similar to murine Tim‐4 macrophages, human CRIg^high^ macrophages are associated with unfavorable prognosis in patients with ovarian cancer [72]. Etzerodt et al. found that CD163^+^Tim4^+^ resident omental macrophages are related to ovarian cancer metastasis [102]. Lyve‐1^+^ mesothelial macrophages promote tumor growth in omentum [103].

Although intestinal resident macrophages, derived from embryonic sources and circulating monocytes, have distinct roles in tissue homeostasis maintenance, they exhibit tumor‐promoting characteristics [11, 127, 128]. Notably, elevated levels of SPP1 and reduced mTORC2 activity in TAMs are associated with a worse clinical prognosis in human colorectal cancer (CRC) patients [105]. FOLR2^+^LYVE1^+^ macrophages have pro‐tumor immunological functions in colorectal tumors and are related to unfavorable prognosis [106].

### 5.2 TRMs Exhibit Antitumor Effects, Associated With a Favorable Prognosis

Tapmeier et al. [89] showed that resident alveolar macrophages maintain key pro‐inflammatory and anti‐tumor gene expression in a mouse model of lung metastasis of melanoma as the disease progresses, while the infiltrating macrophages change from the initial pro‐inflammatory characteristics to the later pro‐tumor characteristics. Furthermore, the proportion of infiltrating macrophages increases as the disease progresses, thus promoting metastasis progression. Wang et al. highlighted that mucosal‐resident alveolar macrophages can exhibit long‐lasting anti‐tumor immunity after influenza exposure, suggesting that inducing trained immunity in TRMs is a promising anti‐tumor strategy [129]. Liver macrophages play a crucial role in restricting growth of certain tumor cells in liver [130, 131]. Li et al. showed that Kupffer cell numbers are less in cancerous tissues than in para‐cancerous tissues and adjacent normal hepatic tissues [132]. Furthermore, research has indicated that bone marrow‐derived cells can replace resident Kupffer cells in transgenic mouse models without impacting liver cancer pathogenesis [13]. Kupffer cells play bimodal role in colorectal tumor metastases to liver [92]. GLUL‐SQSTM1‐RTM was found to act as a positive regulator of immunity in pancreas cancer [96]. Joshi et al. found that TIM4 + LPMs contribute to immune surveillance of the peritoneum at early stages of tumorigenesis [104]. Furthermore, CD169+ macrophages subpopulation originates from tissue self‐renewal and blood stem cells, and depends on vitamin A for development. These macrophages reside in the colonic lamina propria and are associated with clinical outcomes in colon cancer patients [133]. Higher numbers of these macrophages in adjacent lymph nodes are correlated with improved survival rates and a positive prognosis in individuals with colon tumors [107]. TAM may include fetal‐derived macrophages with inherent tumoricidal properties in breast cancer [123, 134]. Unlike in colorectal cancer, Ramos et al. [99] discovered a specific FOLR2 TRMs in primary breast cancer tumors, whose abundance is associated with enhanced patient survival.

## 6 TRMs and Cancer Treatment

The multifaceted roles of TRMs in cancer make it a promising target for cancer treatment. Recent studies [68] have combined macrophage‐targeting agents with established treatments, such as chemotherapy or immunotherapy. Jing et al. reported that cancer treatment may lead to macrophage depletion [135]. Various therapeutic strategies targeting macrophages commonly involve reducing macrophage numbers through inhibition of the CSF1‐CSF1R or the CCL2‐CCR2 pathway [136, 137]. Notably, the FDA‐approved CSF1R inhibitor PLX3397 (pexidartinib) for tenosynovial giant cell tumor treatment has attracted much interest in developing novel small‐molecule CSF1R inhibitors and exploring their potential applications [138].

Various studies have explored alternative molecular targets. Wang et al. [139] outlined several strategies for targeting tumor‐resident myeloid cells, including altering their composition within the TME, blocking immune‐suppressive functions, inducing pro‐inflammatory properties, modulating via cytokines, exploring myeloid cell therapies, and investigating emerging targets, such as Siglec‐15, TREM2, MARCO, leukocyte immunoglobulin‐like receptor (LILR) B2, and common lymphatic endothelial and vascular endothelial receptor 1 (CLEVER‐1). Other factors, such as M‐CSF, CSF‐2, and GM‐CSF, also influence macrophage differentiation, with different stimuli leading to distinct macrophage phenotypes [140]. Sheng et al. [141] indicated that Kupffer cell‐specific targeting is a novel immunotherapy approach for treating HCC, which enhances T‐cell response, reduces liver tumor growth, and sensitizes tumors to anti‐PD‐1 treatment. Additionally, Liu et al. [142] demonstrated that engineered bacteria can overcome Kupffer cell dysfunction and achieve significant therapeutic effects against various types of metastatic liver cancer in mice. TREM2 is highly expressed in TAMs in numerous human tumors [143]. Genetic ablation of Trem2 can enhance anti‐PD‐1 efficacy by reducing immunosuppressive TAMs in murine tumor models [144]. Preclinical studies have shown that FOLR2 specifically expressed on M2 [145] can reduce immunosuppressive function, increase CD8+ T‐cell infiltration, and inhibit tumor growth without inducing toxicity [146]. These findings indicate that FOLR2 can distinguish between immunosuppressive and non‐immunosuppressive TAM subpopulations, offering a potential strategy for identifying and reprogramming immunosuppressive cells in cancer treatment [147]. Staiano et al. [148] discovered that the activation of cannabinoid receptors in lung TAMs can modulate vascular remodeling within tumors by suppressing the secretion of factors that promote angiogenesis and lymphangiogenesis. Furthermore, van Elsas et al. identified a subset of CD163^hi^ TRMs responsible for primary and secondary resistance against T‐cell‐based immunotherapies in mouse models [149]. Therapeutic irradiation is widely used to treat CNS tumors. Studies have demonstrated that various changes occur in the immune response following irradiation, including alterations in resident microglia and infiltration of peripherally derived macrophages [150]. Meng et al. also showed that TAMs depletion using clodronate liposomes before radiotherapy enhances the anti‐tumorigenic effects of ionizing radiation [151]. Rietkötter et al. demonstrated that bisphosphonate zoledronic acid can reduce microglia‐assisted invasion of cancer cells in the brain, thus preventing cerebral metastasis [152].

Macrophage targeting may involve exploiting their plasticity by converting immunosuppressive into immunoreactive macrophages, as demonstrated by the success of targeting CD47‐SIRPα pathway [136]. Although monocyte‐derived macrophages are highly plastic, TRMs exhibit limited plasticity, likely due to the need to maintain tissue homeostasis over extended periods [153]. Wu et al. selectively targeted monocyte‐derived TAMs in mice, resulting in compensatory expansion of tissue‐resident TAMs, possibly enhancing anti‐tumor immunity [154]. Therefore, focusing on specific subsets rather than all macrophages may inhibit the pro‐tumorigenic functions of certain TAM subpopulations while preserving the homeostatic functions of others, thus potentially improving disease outcomes [155, 156]. Besides, reprogramming resident macrophages within human tumors presents challenges, suggesting that depletion rather than repolarization can be a more effective strategy for targeting these macrophages [72]. Additionally, Sung et al. found that ablation tissue‐resident macrophages mitigate cisplatin‐induced ototoxicity and nephrotoxicity in mice [157].

## 7 Interaction Between TRMs and Other Cells in Cancer

**FIGURE 2:**
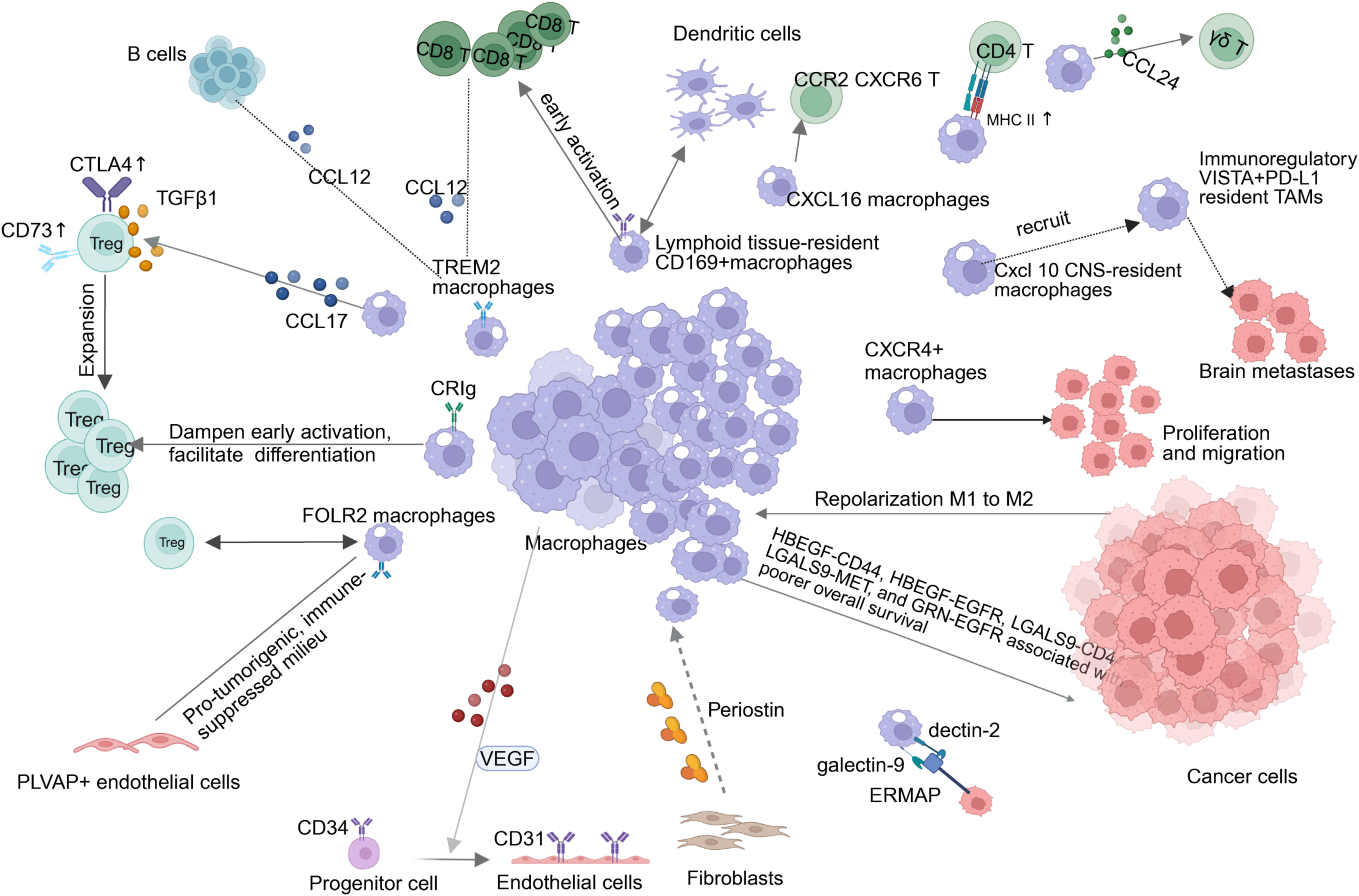
Interactions of tissue‐resident tumor‐associated macrophages with other cells in tumor microenvironment.

The identity and functionality of macrophages are shaped within TME or “niche” [158]. TRMs communicate with cancer cells, various immune cells, and matrix cells within the TME (Figure 2). Through these interactions, TRMs contribute to the complex crosstalk that characterizes the TME, influencing both immune responses and tumor progression.

Cancer cells and TRMs can mutually influence each other. Symchych et al. discovered that a growing tumor can manipulate the functioning of macrophages at a systemic level. They also observed that macrophages in mice with transplanted Ehrlich carcinoma gradually shift in polarization to M2‐like phenotype [159]. Innate lymphoid cells assist resident macrophages in enhancing mucus immunity against cancer cells in non‐small cell lung cancer (NSCLC) [160]. Yang et al. identified various ligand–receptor pairs connecting tumor cells and macrophages, such as HBEGF‐CD44, HBEGF‐EGFR, LGALS9‐CD44, LGALS9‐MET, and GRN‐EGFR. These pairs are associated with poorer overall survival in patients with pancreatic ductal adenocarcinoma (PDAC) [161]. Galectin‐9 and transmembrane receptor dectin‐2, both on Kupffer cells, formed a bridging complex with ERMAP (erythroid membrane‐associated protein), expressed on various cancer cells, to induce the detection and phagocytosis of cancer cells by Kupffer cells. Patients with low expression of ERMAP on tumors had more liver metastases [162].

TRMs and T cells have complex interactions. Tumor‐infiltrating microglia exhibit T‐cell suppressive capacity [15]. Ly6C^low^ F4/80 macrophages, residing outside TME in PDAC, regulate the infiltration of T cells and establish a region of immune privilege [163]. TREM2 TAMs co‐express various factors, such as Arg1, Gpnmb, or Spp1, to facilitate tumor immune evasion [110]. CXCR4^+^ macrophages participate in the process of extracellular matrix remodeling and promote tumor proliferation and migration in a murine orthotopic PDAC model through SPARC secretion via the CXCR4/PI3K/Akt pathway [97]. Guldner et al. identified a subgroup of Chemokine (C‐X‐C motif) ligand (Cxcl) 10‐producing CNS‐resident macrophages that can recruit immunoregulatory VISTA^+^PD‐L1 (programmed cell death ligand 1)‐resident TAMs, thereby promoting tumor evasion in patients with brain metastases [164]. Sepsis‐trained resident macrophages released a chemokine network including CXCL16 triggers tissue residency of T cells via CCR2 and CXCR6 stimulations responsible for decreased risk of tumor development after sepsis cure [165]. Casanova‐Acebes et al. found that tumor‐associated TRMs are involved in antigen presentation to CD4 T cells due to the upregulation of MHC II gene in early NSCLC. This may contribute to early Treg cell expansion due to high levels of *Ccl17* and *Tgfb1*, and impact differentiated Treg cells because of uniquely enhanced CD73 and CTLA‐4 expression compared with BMDMs [86]. Yuan et al. discovered that TRMs express CRIg, inhibit early T‐cell activation, and facilitate differentiation of Foxp3‐regulated (Treg) cells [166]. FOLR2^+^TAM cells, originating from either monocytes or embryonic tissues, exhibit immunosuppressive interactions with Tregs in HCC [75]. Furthermore, lymphoid tissue‐resident CD169 macrophages, as antigen‐presenting cells, play an essential role in the early activation of tumor antigen‐specific CD8 (+) T cells [167]. These macrophages also collaborate with dendritic cells (DCs) to establish self‐tolerance and immune reactions against cancer [168], thereby enhancing anti‐tumor immunity and benefiting survival in bladder cancer [169]. Tertiary lymphoid structures (TLSs) TIM4^+^ macrophages are enriched in immune‐reactive tumors and exhibit microsatellite instability and high infiltration of CD8^+^ T cells [170]. Alveolar macrophages release CCL24, inhibit γδT cells, and impede anti‐tumor response [171].

Besides cancer cells and T cells, TRMs are related to other cell types. For instance, the differentiation of CD34^+^ cells to CD31^+^ endothelial cells within the adventitial vasculogenic zone are activated by macrophage‐derived VEGF [172]. Extracellular matrix cancer‐associated fibroblasts (eCAFs) are correlated with M2 macrophages through the expression of periostin (POSTN) [173]. A subset of macrophages originating from fetal sources in HCC engage in specific communication with endothelial cells bearing similarities to fetal liver endothelium [75]. Moreover, Xiang et al. showed that TAM‐FOLR2 is enriched in early‐stage lung adenocarcinoma, possibly due to the transformation of alveolar resident macrophages and the recruitment of DCs and other TAMs [174]. Che et al. unraveled the complex cellular interplay between apical membrane cells with resident macrophages underlying drug resistance in gastric cancer [175]. The interaction between FOLR2^+^ macrophages and PLVAP^+^ (plasmalemma vesicle‐associated protein) endothelial cells generates pro‐tumorigenic, immune‐suppressed milieu known as an “onco‐fetal ecosystem.” Similar interactions may also be identified in other types of tumors [75, 176].

## 8 Internal Heterogeneity of TRMs

**FIGURE 3:**
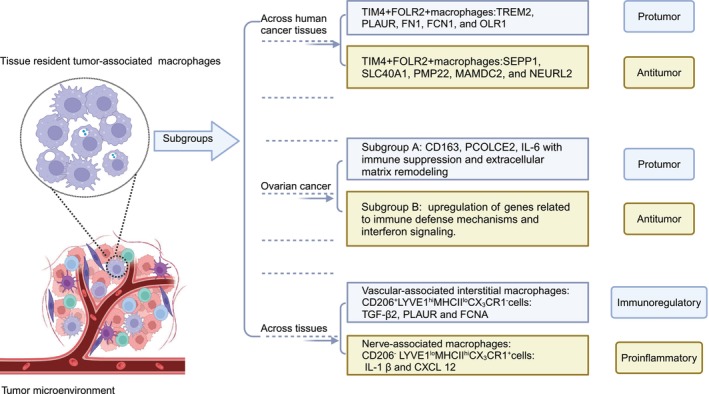
High heterogeneity of tissue‐resident macrophages in tumor microenvironment.

The heterogeneity of TRMs can reasonably account for their multifaceted roles in tumors (Figure 3). These macrophages reside within specialized tissue microenvironments, termed niches, which influence their phenotypic diversity [177, 178]. The differentiation of TAMs and their subsequent heterogeneity are primarily determined by antigen presentation to CD4 T cells [179]. Bugatti et al. uncovered two TIM4^+^FOLR2^+^ clusters, ^CA^TIM4^+^ macrophages (TIM4 expressed on a fraction of cavity macrophages) associated with poor patient survival, and ^TLS^TIM4^+^ macrophages which improve the patients prognosis [170]. In ovarian cancer patients, subgroup A macrophages in ascites showed elevated levels of pro‐tumor markers (CD163, PCOLCE2, procollagen C‐endopeptidase enhancer 2, IL‐6), while subgroup B exhibited decreased levels of markers that promote tumor growth, expression of genes associated with interferon signaling and suppresses the immune function [180]. Moreover, in ovarian cancer patients, CRIg^high^ cells have been found to be resident peritoneal macrophages [23, 181]. Zhang et al. identified LYVE1^hi^ MHC II^lo‐hi^ CX_3_CR1gfp^lo/−^ and LYVE1^lo/−^ MHC II^hi^ CX_3_CR1gfp^hi^ macrophages in the mesenteric and parietal mesothelial linings of the peritoneum [103]. CD206^+^LYVE1^hi^MHCII^lo^CX_3_CR1^−^ cells are considered as vascular‐associated interstitial macrophages which express TGF‐β2, PLAUR and FCNA, suggesting that they may possess immunoregulatory properties. On the other hand, the CD206^−^LYVE1^lo^MHCII^hi^CX_3_CR1^+^ cells associated with nerve bundles or endings are classified as nerve‐associated macrophages. They possess proinflammatory and antigen‐presenting effects by expressing IL‐1β and CXCL12 [182, 183, 184, 185]. Matusiak and colleagues [30] identified spatially segregated TRM niches labeled IL4I1, FOLR2, LYVE1, and MARCO in benign colon and breast tissues. Additionally, IL4I1 marks phagocytosing macrophages and high levels of IL4I1^+^TAMs may reflect good patient outcomes in CRC. Among the solid organs of the body, the highest population of TRMs reside in the liver [186]. Recent studies have identified two distinct subsets of intrahepatic CD68 macrophages, which exert pro‐inflammatory and immunoregulatory effects. A specific subtype of suppressive TAMs which express MARCO has been identified [187]. Evidence suggests that the TRMs promote early dissemination of multiple myeloma by regulating the effects of IL‐6 and TNFα. In a murine myeloma model, CD169+ radiation‐resistant TRMs enhanced early dissemination and progression of myeloma [188]. Moreover, TAMs co‐expressing C1q, HLA‐DR, and APOE were associated with T‐cell exhaustion, which are regarded as tolerogenic and immunosuppressive macrophage populations found in healthy and tumor tissues. These cells are correlated with unfavorable outcomes in various cancers [189].

## 9 Conclusions

Recent research has demonstrated the involvement of TAMs in the progression and metastasis of tumors. In this review, we emphasize the significance of distinguishing TRMs from monocyte‐infiltrated macrophages within the TAM population and discuss the latest proposed methods. In addition, the origin, renewal, self‐maintenance, and biomarkers of TRMs are discussed. A comprehensive analysis of the multifaceted roles of TRMs in tumor development, metastasis, recurrence, and patient prognosis across various tumor sites is presented. Several strategies for targeting TRMs in cancer therapy are discussed. The interactions of TRMs with other cell types in the TME and the heterogeneity of TRMs influence their diverse functions in tumors. By synthesizing current knowledge regarding TRMs, this review enhances our comprehension of multifaceted roles of TRMs and highlights their potential as therapeutic targets in cancer treatment.

## Author Contributions

**Jianhua Chi:** conceptualization (lead), investigation (lead), software (lead), visualization (lead), writing – original draft (lead), writing – review and editing (equal). **Qinglei Gao:** supervision (lead). **Dan Liu:** funding acquisition (lead), project administration (lead), writing – review and editing (lead).

## Ethics Statement

The authors have nothing to report.

## Conflicts of Interest

The authors declare no conflicts of interest.

## Data Availability

The authors have nothing to report.
